# Variations in Shape-Sensitive Restriction Points Mirror Differences in the Regeneration Capacities of Avian and Mammalian Ears

**DOI:** 10.1371/journal.pone.0023861

**Published:** 2011-08-31

**Authors:** Maria Sol Collado, Joseph C. Burns, Jason R. Meyers, Jeffrey T. Corwin

**Affiliations:** 1 Department of Neuroscience, University of Virginia School of Medicine, Charlottesville, Virginia, United States of America; 2 Department of Biomedical Engineering, University of Virginia School of Medicine, Charlottesville, Virginia, United States of America; 3 Neuroscience Graduate Program, University of Virginia School of Medicine, Charlottesville, Virginia, United States of America; University of Auckland, New Zealand

## Abstract

When inner ear hair cells die, humans and other mammals experience permanent hearing and balance deficits, but non-mammalian vertebrates quickly recover these senses after epithelial supporting cells give rise to replacement hair cells. A postnatal decline in cellular plasticity appears to limit regeneration in mammalian balance organs, where declining proliferation responses are correlated with decreased spreading of supporting cells on artificial and native substrates. By culturing balance epithelia on substrates that differed in flexibility, we assessed spreading effects independent of age, showing a strong correlation between shape change and supporting cell proliferation. Then we made excision wounds in utricles cultured from young and old chickens and mice and compared quantified levels of spreading and proliferation. In utricles from young mice, and both young and old chickens, wounds re-epithelialized in <24 hours, while those in utricles from mature mice took three times longer. More cells changed shape in the fastest healing wounds, which accounted for some differences in the levels of proliferation, but inter-species and age-related differences in shape-sensitive restriction points, i.e., the cellular thresholds for shape changes that promote S-phase, were evident and may be particularly influential in the responses to hair cell losses *in vivo*.

## Introduction

Humans and other mammals are vulnerable to permanent hearing and balance deficits that can arise when hair cells are killed by loud sounds, drugs, infections, and other causes. In contrast, fish, amphibians, reptiles, and birds produce hair cells throughout life and recover from such deficits after epithelial supporting cells give rise to replacement hair cells (reviewed in [1], [2], [3], [4]). The cochlea in mammalian embryos can repair limited hair cell losses [5], and mature balance organs from humans and rodents respond to hair cell injury with limited cell replacement, but the repair processes in mammalian ears are minimal compared to the regeneration that naturally occurs in non-mammals [6], [7].

When balance epithelia from neonatal rodents are isolated and cultured with particular exogenous growth factors, supporting cells proliferate robustly; however, their proliferative responses decline progressively in the weeks after birth [8], [9], [10], [11]. That decline strongly correlates in time and magnitude with a progressive postnatal decline in the propensity for sheets of rodent balance epithelia to spread as explant cultures [12]. Contrasting with this, balance epithelia explanted from hatchling and adult chickens spread readily and equally, exhibiting high levels of cell proliferation without any age-related decline [13]. Thus, spreading and proliferation within isolated sheets of balance sensory epithelia appear to mirror the lifelong capacity for hair cell regeneration that occurs in birds and its effective absence in postnatal mammals. In correlation with their divergent spreading capacities, supporting cells in postnatal mammals grow unusually thick circumferential F-actin belts, which eventually occupy 89% of the average cell's cross-section at the apical junction level, while circumferential belts in avian supporting cells remain thin throughout life [13].

More recent experiments tracked supporting cells as they changed shape and quickly closed excision wounds while on their natural substrates in balance organs from late embryonic mice [14], providing quantitative evidence for a tight correlation between the magnitude of supporting cell shape changes and levels of S-phase entry on native substrates, similar to the relationships that have been reported from cell cultures on artificial substrates [15], [16], [17], [18]. However, in balance organs from P14 and older mice, equivalent wounds remained open for at least 48 hours [14]. Therefore, questions persisted as to whether and how wounds in mature mammalian vestibular epithelia might close if allowed to heal longer as well as how epithelia from young and old chicken ears would respond to equivalent wounding while on their natural substrates. Also, it remained to be determined whether experimental manipulations in cultured mammalian vestibular epithelia would show a linkage between cellular shape change and proliferation that is independent of the age of the tissue.

To investigate those questions, we isolated utricular epithelia from embryonic mice, culturing some epithelium sheets on rigid substrates and others on more flexible substrates to promote or limit cell shape change, then we measured the levels of S-phase entry that resulted. In addition, we explanted utricles into organ culture from young and old chickens and mice, made reproducible excision lesions in their sensory epithelia, and then quantified wound closure rates, supporting cell shape changes, and levels of proliferation as the epithelial cells responded to wounds while on their natural substrates.

By culturing vestibular epithelia from a single stage of development under conditions that promote or inhibit cell spreading, our experiments showed that the incidence of S-phase entry is tightly correlated with the magnitude of cellular shape change. In other experiments, we found that wounds in utricles from adult mice took three times as long to close as those in utricles from young mice and those in utricles from hatchling and adult chickens, consistent with the hypothesis that differences in cellular resistance to shape change contribute to the divergent regenerative capacities of mammalian and non-mammalian hair cell epithelia. Also, we observed that considerably fewer cells participated in wound closure in the utricles from adult mice, which may be at least partially attributable to increased resistance to cellular shape change that is hypothesized to accompany the unique postnatal reinforcement that occurs at mammalian supporting cell junctions. Yet, when we measured and compared cellular spreading that preceded S-phase entry in the murine and avian utricles, we found that supporting cells in utricles from adult mice reach high probabilities of entering S-phase only after they have made much more substantial changes in shape than are required for proliferation of supporting cells from avian or neonatal murine ears. The results identify and provide evidence pertaining to two phenomena that help to limit the regeneration of hair cells in mammalian balance organs: biophysical changes that occur as mammalian supporting cells mature postnatally and progressive changes in the shape-sensitive restriction points that must be passed for supporting cells to re-enter the cell cycle.

## Results

### Cellular shape change controls proliferation in the murine macula

Several studies have established a correlation between supporting cell shape change and S-phase entry in murine balance epithelia [12], [14], but these studies did not manipulate supporting cell spreading to test whether it is truly a prerequisite for S-phase entry in supporting cells. To determine whether inhibiting the spreading of supporting cells would result in decreased S-phase entry in embryonic balance epithelia, we used thermolysin to delaminate the utricular epithelium, which consists of both the sensory epithelium (the macula) and the non-sensory epithelium, from E18 mice and explanted those sheets of epithelium onto coverglasses that we had pre-coated with one of three different substrates: poly-L-lysine and fibronectin (PLFN), a thin layer of Matrigel on top of PLFN, or a thick droplet of Matrigel on top of PLFN. Thick droplets of extracellular matrix material (ECM) on coverglasses form flexible gels that are several orders of magnitude less rigid than thin layers of ECM [19], [20], [21], and their flexibility can limit the generation of tension and the spreading of cells [20], [22].

**Figure 1:**
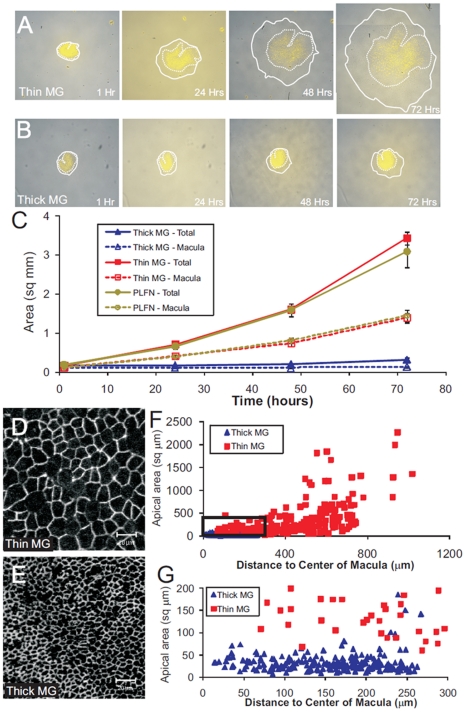
Maculae from E18 mouse utricles spread on thin layers of Matrigel, but spread little on thick droplets of Matrigel. (A) A delaminated utricular epithelium from an E18 mouse utricle cultured on a thin layer of Matrigel (thin MG) shows significant spreading of both the macula (demarcated by the dashed line) and the non-sensory epithelium (tissue between dashed and solid line) over 72 hours in culture. To distinguish the macula from the non-sensory epithelium, hair cells were selectively labeled with the styryl dye, FM1–43 (yellow; [55]). (B) A delaminated utricular epithelium from an E18 mouse utricle cultured on a thick droplet of Matrigel (thick MG) shows little spreading of the macula over 72 hours in culture, though there was spreading of the non-sensory epithelium. (C) The mean total area of the explanted utricular epithelium (solid line) and macula (dashed line) are plotted at each time point for epithelia from E18/19 mouse utricles plated on thick MG, thin MG, or a coverglass coated with poly-lysine and fibronectin (PLFN). (D–E) Confocal images taken near the center of the macula from an E18 mouse utricular epithelium cultured on thin MG (D) or thick MG (E) for 72 hours, and immunostained with antibodies to the tight junctional protein, occludin, to label apical borders of supporting cells. (F) Quantification of the apical area of 210 cells from a single epithelium cultured for 72 hours on thick MG (blue triangle) and 175 cells from a single epithelium cultured on thin MG (red square). Cells grown on thin MG have a significantly larger mean apical area than cells grown on thick MG (384.0±29.7 µm^2^ versus 32.6±1.5 µm^2^; p<0.05, Student's t-test). (G) Magnification of the boxed region in (F) showing that even at the center of the macula, cells cultured on thin MG have an enlarged apical area compared to cells cultured on thick MG.

The utricular epithelia that we cultured on thin Matrigel expanded in area by nearly 20-fold during the 72-hour culture period (area increased 1958%±86%, n = 5, Fig. 1A,C). The sensory epithelium at the center of the utricular epithelia increased in area by 1097%±178% (Fig. 1A,C). Thus, epithelial spreading occurred in both the sensory epithelium and in the non-sensory epithelium that surrounds it. The epithelia that we cultured on glass coated with only PLFN showed similar spreading (n = 3; Fig. 1C). In contrast, the sheets of epithelia that we cultured on thick, flexible Matrigel increased in area just 75%±18%, and the macula in the center of each increased on average by only 17%±11% (n = 4; Fig. 1B,C).

Our measurements showed that the mean apical area of cells within the macula of sheets cultured on thin Matrigel was 11 times greater than the mean area of cells in the sheets that were cultured on thick Matrigel (384.0±29.7 µm^2^ versus 32.6±1.5 µm^2^; n = 176 and 210 cells; Student's t-test, p<0.05). In the sheets cultured on thin Matrigel, the magnitude of cellular shape changes increased with increasing distance from the center of the macula. In contrast, cell areas within the macula in the sheets cultured on thick Matrigel varied little. Yet, the non-sensory epithelium at the periphery of the sheets cultured on the thick Matrigel did spread (Fig. 1F,G), demonstrating that the flexibility of the thick Matrigel had an effect that was particularly limiting to shape change by supporting cells in the macula.

**Figure 2:**
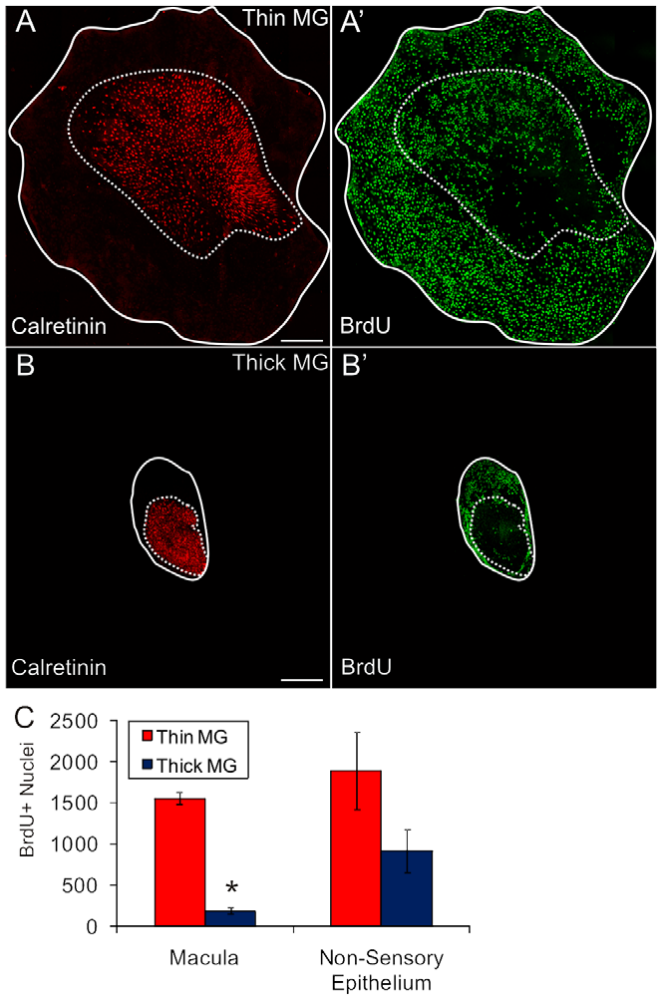
Cells within spread maculae enter S-phase. (A–B) Z-projected confocal image stacks of delaminated utricular epithelia from E18 mice immunolabeled with antibodies for the hair cell marker, Calretinin (red) after being cultured on thin (A) or thick (B) Matrigel for 72 hours. BrdU (green) immunolabeling is shown in A′ and B′. BrdU+ nuclei are largely restricted to the non-sensory epithelium on the thick Matrigel, but BrdU+ nuclei are present throughout the macula (edges marked with dashed line) and non-sensory epithelium (edges marked with solid line) after spreading on thin Matrigel. Scale bars, 300 µm. (C) Quantification of the number of BrdU+ nuclei in maculae and non-sensory epithelia from cultures on thick MG and thin MG is shown (n = 4). There is a significant increase in the number of BrdU+ nuclei within the sensory epithelium of cultures grown on thin MG compared to cultures grown on thick MG (p<0.0001; Student's t-test).

When we cultured epithelium sheets in BrdU containing medium on thin Matrigel, that resulted in many BrdU+ nuclei scattered throughout the macula (1556±74 per E18 macula; n = 4), whereas maculae in the sheets which were cultured on thick Matrigel that inhibited supporting cell spreading contained relatively few (182±33 per macula; n = 4; Fig. 2). Thus, differences in the amount of shape change that supporting cells from utricles of the same age undergo appear to determine the relative likelihood for those supporting cells to pass through the restriction point and enter S-phase. Appreciable numbers of BrdU+ nuclei were observed within the non-sensory epithelium on both thin and thick Matrigel, showing that both substrates can support high levels of epithelial cell proliferation (Fig. 2). These results demonstrate that cellular shape changes and/or substrate rigidity are prerequisites for supporting cells to pass the restriction point and enter S-phase.

**Figure 3:**
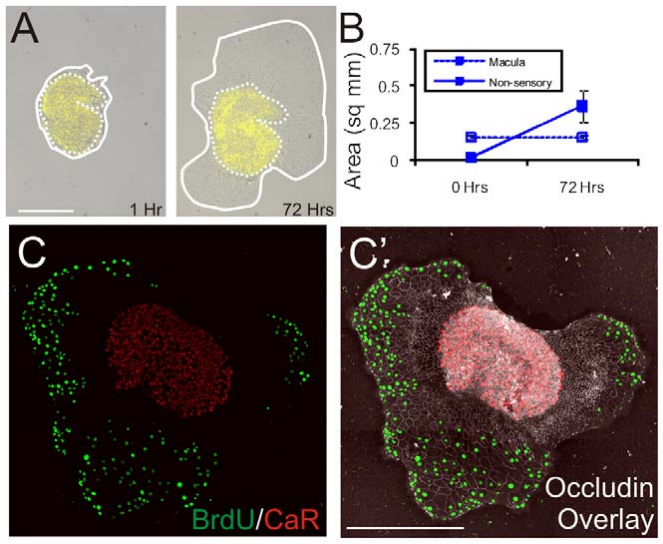
Cells from the macula of 2-week-old mouse utricles lose the ability to change shape and enter S-phase. (A) Images of a delaminated utricular epithelium from a P15 mouse cultured for 72 hours on a thin layer of Matrigel taken 1 hour after plating (left) and after 72 hours in culture (right). The epithelium was preincubated in FM1–43 to label the hair cells (yellow), and both the edge of the utricular epithelium (solid line) and macula (dashed line) are marked. (B) Quantification of the total area of utricular epithelia and maculae from P15 mice cultured for 72 hours on thin Matrigel. Although the total area of utricular epithelia increases over 72 hours, the area of maculae remains unchanged (101%±9% of initial size after 72 hours; n = 5). (C) Z-projected confocal image stack of a delaminated utricular epithelium from a P15 mouse cultured for 72 hours on thin Matrigel and immunolabeled for BrdU (green) and the hair cell marker, Calretinin (red). An α-occludin overlay (C′) shows the extent of cell shape change in the non-sensory epithelium. All BrdU+ nuclei were in the spread regions of the non-sensory epithelium. The macula did not spread or contain BrdU+ nuclei.

When epithelia from P15 mouse utricles were cultured on thin Matrigel the macula regions at their centers increased in area only 1%, with none of the supporting cells incorporating BrdU. Non-sensory cells in the same sheets readily changed to spread shapes, however, and many became BrdU+ (n = 5, Fig. 3). These results help to differentiate between the potential effects of substrate rigidity and changes in cellular shape, since P15 supporting cells that did not change shape also failed to enter S-phase even after culturing on a rigid substrate that permitted many cells to change shape and proliferate in the surrounding non-sensory epithelium. Consistent with the hypothesized effect of the maturational reinforcement of their junctional cytoskeletons [13], the more mature (P15) supporting cells appeared more resistant to changing from columnar to spread cell shapes.

### Wounds close rapidly in utricles from young and old chickens

**Figure 4:**
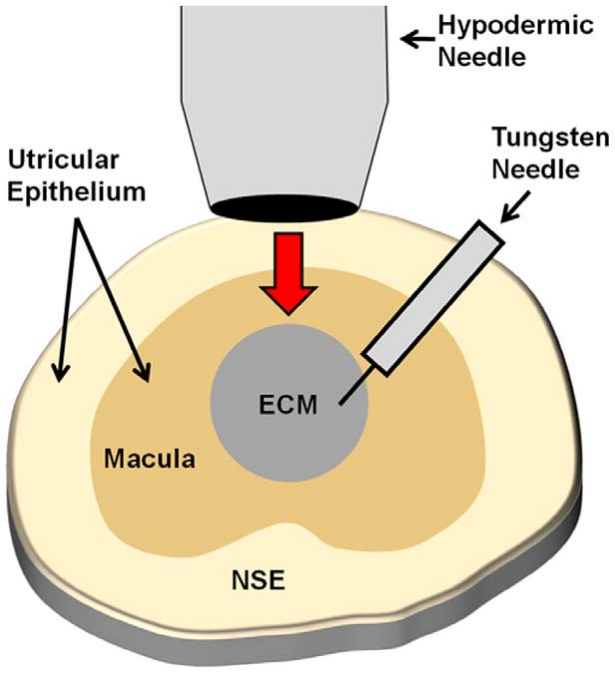
Wounding assays in the macula of the gravity-sensitive utricle. Located centrally in the utricular epithelium is the macula (dark tan), which is made up of a single layer of epithelial cells called supporting cells intermixed with sensory hair cells. The non-sensory epithelium (NSE, light tan) surrounds the macula and is comprised of just non-sensory epithelial cells. These epithelial cells reside on their native extracellular matrix, which is in turn anchored by underlying stromal cells. For wounding assays, an electro-polished hypodermic needle is pressed into the epithelium at the center of the macula, and the cells within the lesioned area are excised with a sharpened tungsten needle exposing the underlying native ECM (gray).

**Figure 5:**
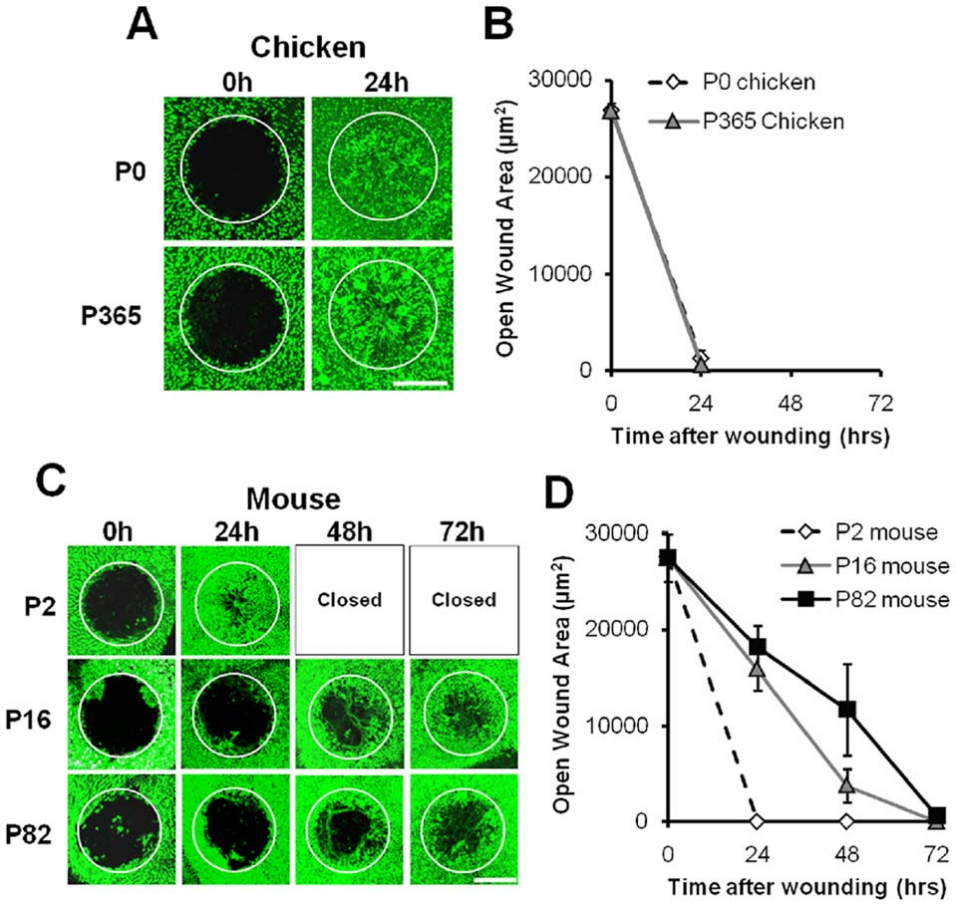
Utricles from chickens and young mice close wounds faster than utricles from older mice. (A) Z-projected confocal image stacks (20×/0.75NA objective) of wounded utricles from P0 and P365 chickens. Utricles were fixed after wounding at the designated time points and labeled with phalloidin (green) to view F-actin in the apical junctions of cells. Wounds are almost closed by 24 hours (initial size of wound shown with white circles). (B) Quantification of open wound areas versus time after wounding in chicken utricles (n = 6 for each age group). (C) Wounds in utricles from P2 mice are completely closed within 24 hours. Wounds in utricles from P16 and P82 mice, however, take 72 hours to close. Scale bars in A and C, 100 µm. (D) Graphs of open wound areas versus time after wounding demonstrate that wound closure times and rates decline with age in mice.

Unlike rodents, sensory epithelia isolated from chicken utricles have been shown to spread and proliferate without any age-related decline when cultured on a rigid, artificial fibronectin substrate [13]. Because age-related modifications to the ECM could affect the capacities for supporting cell shape change and proliferation in avian utricles that mature *in vivo*, we investigated the spreading and proliferation of avian supporting cells on their native ECM substrate by making excision wounds in the macula of whole mount utricles that we dissected from young and adult chickens (Fig. 4). Those wound areas became 95% and 98% re-epithelialized by 24 hours in the utricles from hatchling (P0) and 1-year-old (P365) chickens, respectively (n = 6; Fig. 5A,B; Table S1). Phalloidin labeling showed that supporting cells maintained their junctions as they changed shape and collectively migrated, closing all the wounds completely in 48 hours (n = 4; Fig. 5A). The results show that avian vestibular supporting cells differ substantially from their counterparts in mammals [14] in that they retain a lifelong and apparently undiminished capacity for responding to epithelium injury by rapidly changing from their normal columnar shapes to spread shapes on their native substrate. These results in chicken utricles are also consistent with expectations based on the lifelong retention of thin circumferential F-actin belts in their supporting cells.

### Wounds in adult mouse utricles close through slower collective migration

In our previous study, balance epithelia from late embryonic (E18) mice closed excision wounds rapidly, while equivalent lesions in utricles from 2-week-old mice remained open after 48 hours [14]. To determine whether and how the supporting cells in mature vestibular organs would eventually change shape and close wounds, we made excision lesions in organ-cultured utricles from juvenile (P16) and adult (P82) mice, and fixed groups of cultured utricles at 24-hour intervals. For comparison, wounds were also made in utricles from young (P2) mice. The wounds in the P2 utricles re-epithelialized the excision area in 16–24 hours (n = 4; Fig. 5C,D; Fig. S1). In the utricles from P16 and P82 mice the rate of closure was much slower than in the utricles from young mice and young and adult chickens. Re-epithelialization covered less than half of the excision area by 24 hours, and complete closure took 72–96 hours (n = 5; Fig. 5C,D; Fig. S1; Table S1).

To determine whether the longer wound closure times in the utricles from older mice might have resulted from a delay in the start of the closure process, we made measurements of open wound area versus time since wounding for the groups of P16 and P82 mouse utricles. The results revealed that mean open wound areas decreased linearly (r^2^ = 0.95 and 0.99, respectively), indicating that the longer closure time in adult epithelia resulted from consistently slower collective migration speeds, not from a delayed start.

### Chicken supporting cells are more proliferative following wound closure than those in mice

**Figure 6:**
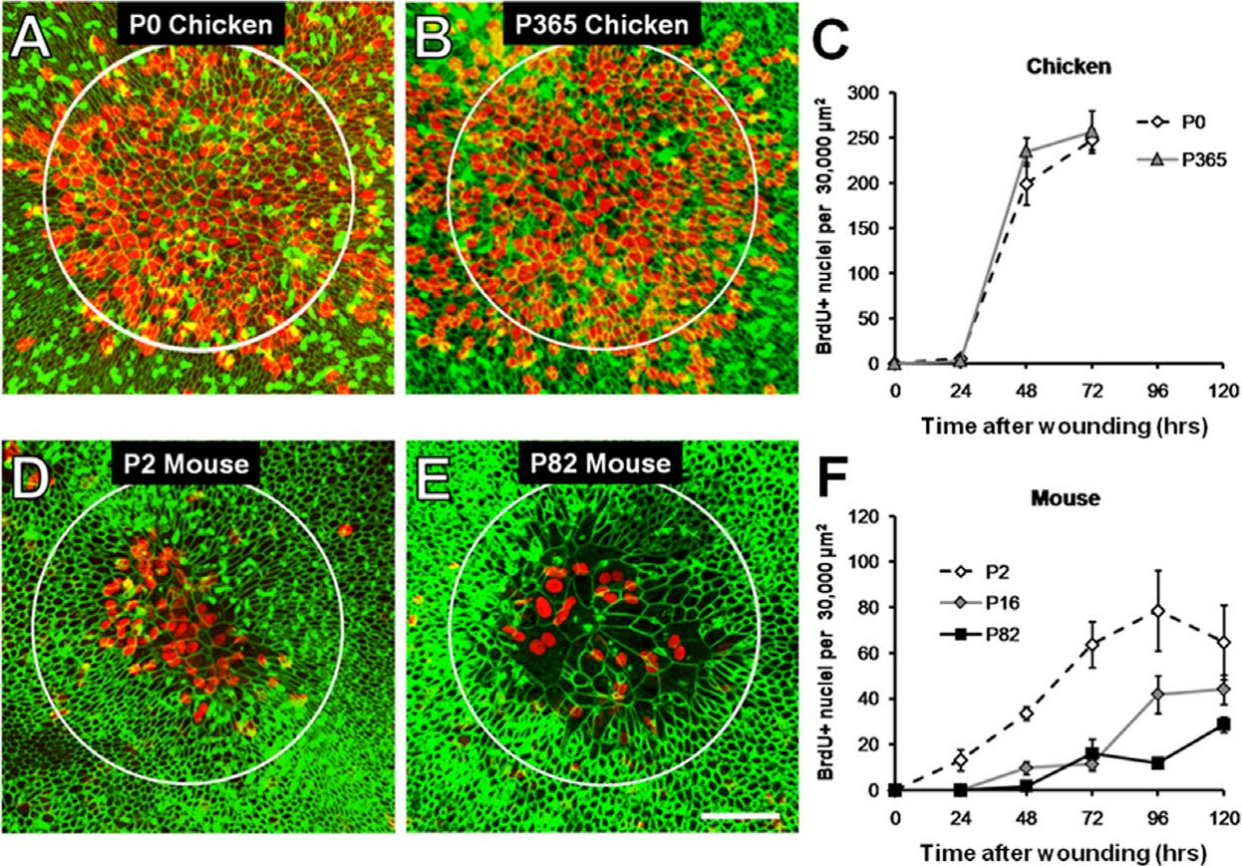
Within closed wounds, more avian supporting cells incorporate BrdU compared to those from mice. (A–B) Z-projected confocal image stacks (20×/0.75NA objective) of the wound area in utricles from P0 and P365 mice fixed after 72 hours of culture (48 hours after wound closure). The apical outlines of supporting cells are visible with phalloidin labeling of F-actin (green). Many supporting cells within the closed wound (initial wound size shown with white circles) have incorporated BrdU (red). (C) Quantification of the number of BrdU+ nuclei in the 30,000 µm^2^ wound area versus time in chickens. The number of BrdU+ nuclei increases between 24 and 48 hours after wounding, then begins to level off by 72 hours. (D–E) Confocal images of the wound area in utricles from P2 and P82 mice taken after 72 and 120 hours of culture, respectively (48 hours after wound closure). Scale bar in D, 50 µm. (F) Quantification of the number of BrdU+ nuclei in 30,000 µm^2^ wound areas as a function of time in P2, P16, and P82 mice. In all age groups, the number of BrdU+ nuclei increases between 24 and 48 hours after wound closure.

Since the balance epithelia spread into the same-sized wounds in utricles from young and old chickens and mice, we could next determine whether wound closure responses would result in similar levels of S-phase entry for the different species and age groups. For this, we fixed groups of utricles at different time points and assayed for nuclei that incorporated BrdU from the culture medium. At 24 hours, the supporting cells in the young utricles from both species had re-epithelialized 95% or more of the wound area, but few had entered S-phase (<13 BrdU+ nuclei per utricle; n = 4–7; Fig. 6; Tables S1, S2), which is consistent with results of isolated epithelium experiments where supporting cell spreading preceded re-entry into the cell cycle [12], [13].

The peak levels of S-phase entry varied between age groups and species. Fully re-epithelialized wound areas in utricles from P0 and P365 chickens contained similar numbers of BrdU+ nuclei, and significantly more than in the closed wounds in all the mouse utricles (n = 4–7; ANOVA with Tukey test, p<0.05; Fig. 6A–C; Table S2). The next highest levels of BrdU labeling were present in the closed wounds in utricles from P2 mice, which contained significantly more than the P16 and P82 utricles (n = 4–9; ANOVA with Tukey test, p<0.05; Fig. 6D–F; Table S2). Peak incidences of BrdU+ nuclei were similar in the P16 and P82 mouse utricles and remained low, even after they were cultured with BrdU for 120 hours after wounding (Fig. 6F, Table S2). Thus, fewer supporting cells enter S-phase in utricles from adult mice than in utricles from young mice and chickens of all ages. Although the supporting cells in utricles from young mice close wounds more rapidly than supporting cells in chickens, their incidence of S-phase entry is 25% of that for chicken supporting cells, which suggests that there are important differences between species in the supporting cells' response to shape change.

### Shape changes alone do not explain the proliferative differences between avian and mammalian utricles

We considered several hypotheses that held the potential to explain the differences we observed in the number of cells that re-entered the cell cycle after wound closure. The four-fold higher number of BrdU+ supporting cells in the avian wound sites could be explained if more supporting cells participated in wound closure in chickens than in mice, but the mean number of cells in the closed wounds in the chicken utricles did not differ significantly from those in P2 mouse utricles (P0 and P365 chicken utricles = 694±46 cells, and 672±26 cells, P2 mouse utricles = 662±22; n = 3 for each; p>0.05, ANOVA with Tukey test). Closed wound areas in utricles from P82 mice contained significantly fewer cells (351±9 cells; n = 3; p<0.05, ANOVA with Tukey test). When the number of BrdU+ cells was expressed as a fraction of the total cells counted within the wounds, it showed that in utricles from young and old chickens more than 33% were BrdU+, while only ∼12% of the cells in the wounds in young mouse utricles were BrdU+, and ∼8% were in the older mouse utricles. The differences in the numbers of cells that moved into the wound areas account for part of the difference in S-phase entry in utricles from young and old mice, but substantial differences in proliferation between chickens and young mice remained and are not attributable to differences in the numbers of cells that move into these lesions.

If avian supporting cells rapidly underwent multiple rounds of division and murine supporting cells did not, that could provide an alternative explanation for the substantial difference in the number of BrdU+ cells in their closed wounds. To test for this, we cultured wounded chicken utricles with colchicine (10 µM) for 72 hours in order to block mitosis. The colchicine-treated utricles contained 132±20 BrdU+ nuclei (n = 8), while matched controls contained 247±33 BrdU+ nuclei (n = 8; Fig. S2). The control cultures in which cells were allowed to complete mitosis had just under twice the number of BrdU+ nuclei as the cultures where mitosis was blocked, which suggests that on average the proliferating cells completed cytokinesis just once during the 72 hour culture period. Therefore, differences in timing and number of cells re-entering the cell cycle between chickens and young mice cannot be explained by the number of cells that participate in the wound closure response or by rapid cycling. Instead, the data suggest that chicken supporting cells proliferate more readily in response to wound closure and shape change than their counterparts in mammals.

**Figure 7:**
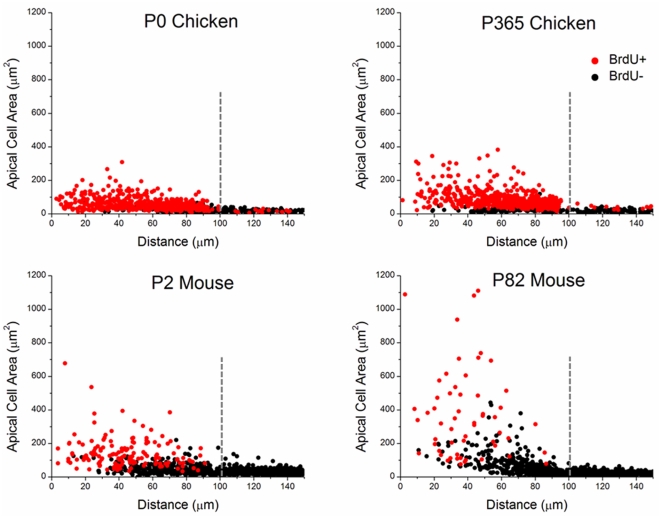
Supporting cells from chickens and mice undergo different degrees of shape change to close wounds. Shown are scatter plots of apical cell areas versus distance to the center of the excision area 48 hours after wound closure (400 cells per utricle for 3 utricles per age group). Apical cell areas were measured out to 150 µm from the center of the excision area (radius of the excision area was roughly 100 µm, shown as a dashed line). If a BrdU+ nucleus fell within the apical area, we recorded that cell as BrdU+ (BrdU+ data points are red circles, BrdU− data points are black circles). In all species and age groups, the largest cells were near the former wound center. There was a clear trend between S-phase entry and increased cell area. Supporting cells from P0 and P365 chickens underwent small shape changes with little variability, but many were BrdU+. Despite undergoing the largest shape changes, comparatively fewer cells from P82 mouse utricles were BrdU+.

We next assessed whether different amounts of cellular spreading could have led to the differences in the amounts of proliferation in the avian and murine wound sites. To make this assessment, we first measured average apical areas of cells in unlesioned control utricles and in sites of closed wounds. Then, we plotted the average areas of cells within closed wounds as a function of distance from the wound center. Apical outlines of supporting cells averaged 12.0 µm^2^±0.4 µm^2^ in unlesioned P0 chicken utricles fixed *in vivo* (n = 130 cells), while those in P365 adults averaged 13.4 µm^2^±0.5 µm^2^ (n = 131). Supporting cells in unlesioned utricles in P2 mice averaged 14.5 µm^2^±0.4 µm^2^ (n = 127 cells), and 18.1 µm^2^±0.4 µm^2^ in P82 adults (n = 123 cells). In closed wounds in P0 chicken utricles fixed at 72 hours, cells near the wound center averaged 67±3 µm^2^, with the widest cell being 308 µm^2^. The mean area decreased to 21±2 µm^2^ for cells located at the outer edge of the original wounds' circumference (Fig. 7, Figs. S3, S4). Utricles from P365 chickens showed a similar pattern, with a mean area of 118±7 µm^2^ near the wound center, and a maximum of 382 µm^2^, while those at the circumference of the original wound averaged 25±5 µm^2^ (Fig. 7, Figs. S3, S4).

Apical cell outlines in P2 mouse utricles fixed 72 hours after wounding were slightly larger than those in chicken wounds, but the center-to-edge gradient was similar (means = 145±14 µm^2^ near the center, 32±2 µm^2^ at the original wound radius, and max = 677.4 µm^2^). In utricles from P82 mice, cell areas near the wound center were approximately twice those in the P2 utricles (mean = 284±36 µm^2^, max = 1100 µm^2^). Since approximately half as many supporting cells covered the wound areas in adult mouse utricles compared to those in chickens and young mice, the adult mouse supporting cells had to become approximately twice as large to cover similar-sized wounds. This may account for some of the differences between adult mice and the other test groups in the incidence of BrdU+ nuclei. However, the average shape changes that P2 mouse supporting cells and chicken supporting cells undergo do not appear to fully account for their differing levels of proliferation.

### Adult murine supporting cells make large changes in shape before entering S-phase

**Figure 8:**
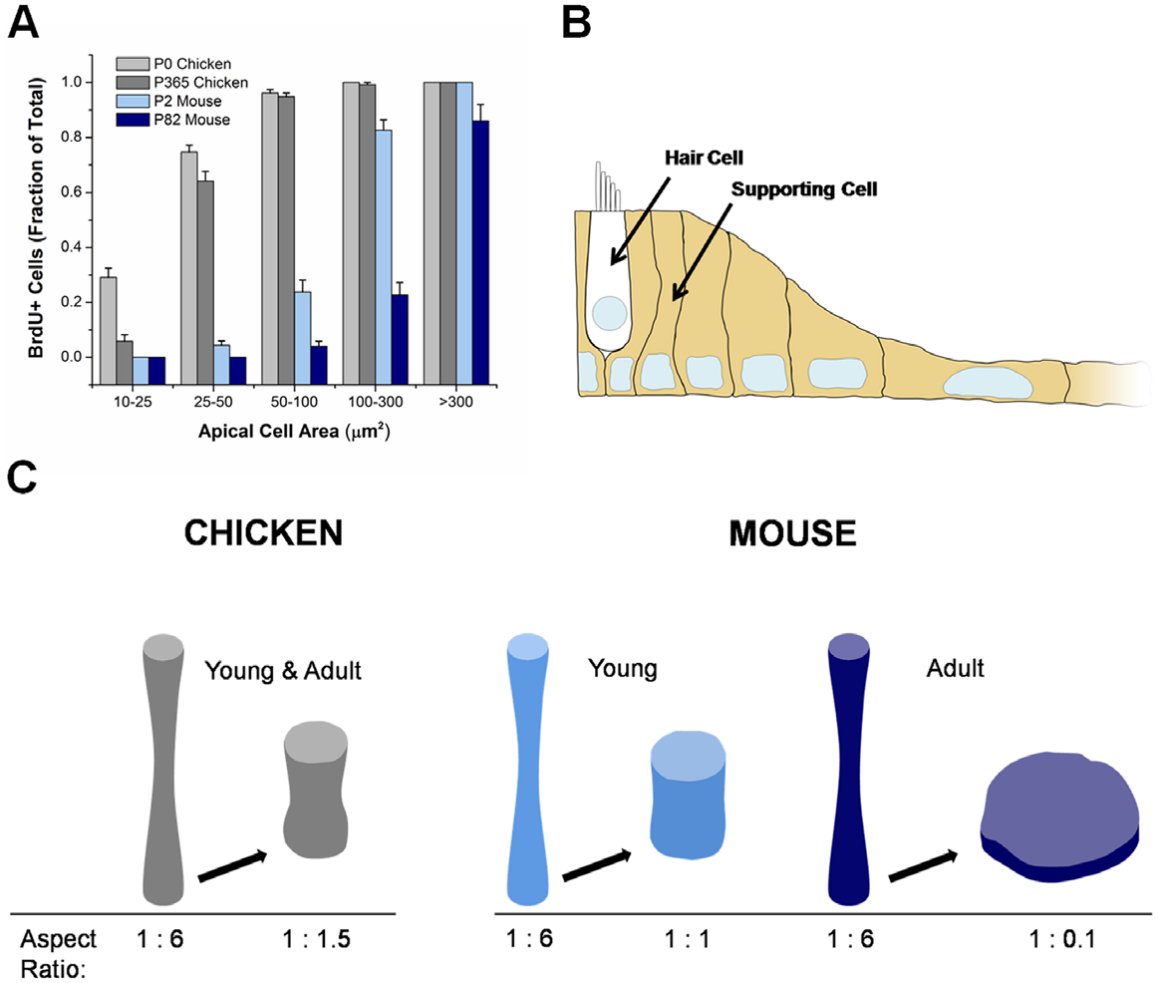
Supporting cells from mice require larger shape changes than those from chickens in order to increase their likelihood of entering S-phase. (A) Graph summarizes the fraction of BrdU+ nuclei for the given range of apical areas of supporting cells within the wound 48 hours after closure (n = 3 utricles per age). The probability of a supporting cell entering S-phase after smaller changes in apical cell area was lower in mice than chickens. Supporting cells from adult mice could reach a high probability for entering S-phase but required larger changes in shape to do so. (B) Cross-sectional schematic of the macula depicting how supporting cells change shape while maintaining cell-cell contacts to close wounds. The supporting cells adjacent to the hair cell have tall, columnar shapes like those they maintain *in vivo*, whereas supporting cells to the right have spread, flat shapes. (C) Schematic diagram comparing the change in aspect ratio of supporting cells from adult chickens and mice that correlates with a >80% probability of entering S-phase. Aspect ratios were obtained for supporting cells *in vivo*, and the change in height was calculated from apical areas by holding cylindrical volume constant. Supporting cells from chickens and young mice have a >80% probability of entering S-phase when they are still columnar or cuboidal, but supporting cells from adult mice have to achieve a highly flattened shape to have similar probabilities.

Scatter plots of the apical areas of BrdU+ and BrdU− supporting cells show that many cells from adult mice spread to large areas but remained BrdU− (Fig. 7, Fig. S3). We analyzed the relationship between apical cell area and incidence of S-phase entry in wounded utricles 48 hours after closure by dividing area data into 5 bins (10–25 µm^2^, 25–50 µm^2^, 50–100 µm^2^, 100–300 µm^2^ and >300 µm^2^) and plotted the percentage of cells per bin that were BrdU+. In utricles from P0 chickens, 29% of the cells with apical areas between 10–25 µm^2^ (i.e. cells with <2 times the mean *in vivo* area) were BrdU+. In P365 chicken utricles, 5.9% of cells in that area bin were BrdU+ (Fig. 8A). Cells of 25–50 µm^2^ had much greater rates of S-phase entry (75% BrdU+ for P0; 64% for P365; Fig. 8A), and nearly all the chicken supporting cells that had spread to >50 µm^2^ were BrdU+ (97% for P0; 96% for P365). These data show that the probability that supporting cells from hatchling and adult chickens will enter S-phase increases sharply when those cells spread to two or more times the mean area of a supporting cell in an undamaged utricle.

In utricles from P2 mice, <23% of the supporting cells with apical areas of 10–25 µm^2^, 25–50 µm^2^, and 50–100 µm^2^ were BrdU+, and when such cells spread to 100–300 µm^2^ their incidence of BrdU labeling increased to 83% (Fig. 8A). In P82 mouse utricles, S-phase entry by supporting cells required even greater shape changes, with only 23% of cells that spread to 100–300 µm^2^ becoming BrdU+. However, when adult cells spread to >300 µm^2^, 86% became BrdU+. We conclude from these data that the supporting cells in wounded utricles from adult mice will reach a high probability for entering S-phase only after making much greater changes in shape than are required to promote high levels of S-phase entry amongst the supporting cells from chickens and neonatal mice.

For both chicken and mouse supporting cells, the mean *in vivo* aspect ratio, expressed as the ratio of apical cell surface diameter to the cell's apex-base height, is approximately 1∶6. Therefore, spreading that increased the mean apical cell area by two-fold would drop the mean cellular aspect ratio to 1∶1.5 (assuming that there would not be rapid changes in cell volume, Fig. 8B,C). In chicken utricles, supporting cells that change aspect ratio by that amount have a 94–96% probability of entering S-phase. In contrast, equivalent changes in the mean aspect ratios for murine supporting cells are correlated with low (23%) probabilities of S-phase entry in P2 utricles, and very low (4%) probabilities in P82 adult mouse utricles. Spreading to a 4-fold greater apical area would change cellular aspect ratio to 1∶1.1, approximately the ratio for a cuboidal cell shape, which is correlated with 83% BrdU labeling for P2 mouse utricles and 23% for P82 utricles (Fig. 8B,C). The results show that supporting cells in adult mouse utricles can reach an 86% probability of entering S-phase by changing to a spread shape, with an aspect ratio of 1∶0.1, at which point the apical outlines of such supporting cells occupy at least twelve times the area occupied by the apical outline of the average supporting cells in undamaged utricles of adult mice *in vivo* (Fig. 8C).

## Discussion

The results provide evidence that the propensity for vestibular supporting cells to enter S-phase is linked to their ability to change from columnar to spread shapes. By culturing murine vestibular epithelia on Matrigel substrates that differed in flexibility we were able to inhibit supporting cell spreading in age-matched samples, which markedly reduced S-phase entry. Our results also help to explain how increased resistance to shape change in mammalian supporting cells could limit cell replacement. On their native substrate, supporting cells from chickens and young mice closed excision wounds three-times faster than the supporting cells of adult mice. The slower closure in adult utricles was coupled with fewer cells migrating into the wounds and undergoing larger deformations to cover the excision area. The differences observed were consistent with the hypothesis that thicker circumferential F-actin belts would contribute greater resistance to cellular deformation, but that hypothesis alone does not account for the all of the observed differences in the levels of S-phase entry. For example, three times as many cells entered S-phase in avian utricles as in neonatal mouse utricles, despite similar mean levels of cellular shape change. Our analysis suggests that inter-species and age-related variations in the thresholds for cellular shape changes that promote S-phase entry may account for the differences in S-phase entry that are not attributable to the differences in cellular resistance to shape change.

### Shape-change and maturation of supporting cells

The diminished spreading of mammalian vestibular supporting cells appears to stem from intrinsic properties acquired as the cells mature postnatally, and not from substrate changes, since age-related declines in spreading occur independent of culturing on poly-L-lysine, fibronectin, laminin, collagen IV, or Matrigel ([12]; Fig. 6). However, loss of integrin activation in supporting cells could potentially contribute to declines in spreading. Crosstalk between adherens junctions and integrins can influence migration and spreading [23], and stabilization of cell-cell and cell-matrix adhesions certainly could act synergistically. In utricles from adult mice, supporting cells distal to a wound edge do not change shape and fail to participate in closure, suggesting that they are more resistant to deformation than their counterparts in younger mice and chickens, which may result from the unusual thickening of the circumferential F-actin belts that occurs as vestibular supporting cells in mammals mature during the first postnatal weeks [13].

As wounds in adult utricles close, small numbers of cells at the wound edge deform greatly. Phalloidin-labeling showed that actin belts in those leading edge cells become relatively thin (Figs. 5,6), suggesting that proximity to a wound edge may lead to rapid cytoskeletal alterations and decreased resistance to change shape. How this occurs remains to be determined, but wound edges are potential sites of membrane disruption and calcium influx [24]. In fact, calcium waves propagate from sites of damage in hair cell epithelia [25], and could destabilize the actin cytoskeleton via calcium-activated severing proteins, such as gelsolin and villin, or may activate motor proteins at the cell's leading edge [26], [27].

### Cellular shape change appears to control S-phase entry in supporting cells

In this study and others, decreases in the capacity for postnatal mammalian supporting cells to change shape have been paralleled by declines in proliferation [8], [10], [12], [14]. Various potential contributors to decreased proliferation have been evaluated, including decreased expression of growth factor receptors and changes in the expression of cyclin D1 and p27Kip1 [9], [28], [29], [30], [31]. In the embryonic mammalian cochlea, changes in cyclin dependent kinase inhibitors exert important regulation over proliferation, but roles in vestibular epithelia remain less clear. The observation that mature vestibular supporting cells re-enter the cell cycle after completing large shape changes (Figs. 6,8) indicates that maturational limits to mammalian supporting cell proliferation can be overcome.

Substrate stiffness can be a potent regulator of cellular shape change, with compliant substrates inhibiting cell spreading, and stiffer substrates promoting it [32], [33], [34]. Cells in turn match the elasticity of their substrate by increasing Rho-mediated contractility when on stiff substrates, which presumably leads to degradation of p27Kip1, increased cyclin D1, hyperphosphorylation of retinoblastoma, and S-phase entry [16], [35]. The differences in the magnitude of cellular shape changes we observed in the matched samples of epithelia we cultured on rigid and more flexible substrates resulted in markedly different levels of S-phase entry, consistent with the hypothesis that cellular shape change is an upstream regulator of proliferation in supporting cells.

Other candidate mechanisms for shape control of proliferation include nuclear volume changes that promote chromatin decondensation [36], alterations in cytoplasmic and nuclear calcium concentrations [37], [38], activation of focal adhesion kinase [39], and regulation via Rho family GTPases [40], [41]. The PI3K-Akt-TOR and ERK/MAPK pathways have been implicated in the control of proliferation in vestibular supporting cells from mammals and birds and could act downstream of signals that originate through changes in shape [42], [43]. Many of these mechanisms are influenced by the tumor suppressor activity of E-cadherin [44], which is absent or expressed at low levels in supporting cells of birds [45], but accumulates at supporting cell-supporting cell junctions in mammalian vestibular and cochlear epithelia in parallel with actin belt reinforcement [10], [46]. In chick utricular epithelium, increased proliferation is dependent upon N-cadherin activation and is correlated with decreased cell density, which is effectively equivalent to increased cell spreading in an intact, pseudo-stratified epithelium [47]. Thus, age- and species-related differences in the cytoskeletal and adhesive components of junctions between utricular supporting cells could control the propensity for these cells to change their shape and respond to shape change by entering S-phase.

### Species- and age-dependent differences in S-phase entry

The differences in absolute levels of proliferation that we observe between chickens and mice do not appear to be explained solely by differences in the numbers of cells that change shape when closing wounds. We found that supporting cells from chickens and neonatal mice are likely to enter S-phase while still maintaining columnar or cuboidal shapes, but more dramatic spreading is required for S-phase entry in supporting cells from adult mice (Fig. 8). These results lead to the hypothesis that supporting cells that have different regenerative capacities require different amounts of shape change before they will pass through their cell cycle restriction points.

Many cells increase proliferation after changing to a spread shape. The shape-sensitive restriction point has been defined as the checkpoint before S-phase that can be passed after cells change shape [16]. The minimal shape change at which cells become responsive has been found to vary by tissue type; for example, rat kidney epithelial cells are less proliferative on 300 µm^2^ and 500 µm^2^ microwells than mammary epithelial cells [18]. The results of our experiments on inner ear cells that remained in situ indicate that shape-sensitive restriction points also can vary between the same cell type in different species and a cell type at different states of postnatal maturity in one species.

Also, higher levels of ongoing S-phase entry occur in undamaged chicken utricles, where the hair cell population turns over every few months [48], [49], [50]. In mouse utricles on the other hand, S-phase entry is already low at P2, and turnover is not believed to occur.

### Implications for inner ear regeneration

In the chicken cochlea, apical supporting cell surfaces quickly expand by roughly three-fold near sites of sound-induced hair cell loss [51], [52], [53], [54]. Such changes in shape are followed by supporting cell divisions that lead to the formation of replacement hair cells and supporting cells. Our analysis of cell proliferation in excision-wounded utricles has shown that when vestibular supporting cells in chicken utricles spread to twice the apical area found in undamaged controls they will have a high likelihood for S-phase entry (Fig. 8). Thus, supporting cells in chickens are likely to enter S-phase when they expand to cover the area left open after one or two hair cells have been lost. Supporting cells in utricles from adult mice, however, are unlikely to pass through their shape-sensitive restriction point except in cases of more extensive hair cell loss (Fig. 8).

In conclusion, cellular shape changes appear to have a critical role in determining whether supporting cells in the balance organs of birds and mammals will proliferate and contribute to regeneration. Shape change precedes and is predictive of subsequent supporting cell entry into S-phase. Also, the magnitude of the shape change that correlates with a high likelihood of transition from quiescence into S-phase varies between supporting cells from birds and mammals, increasing significantly with postnatal age in mammals. Although the supporting cells in adult mammalian utricles normally appear refractory to shape change and proliferation, our results show that these cells can enter S-phase when they happen to be near a wound edge and respond to it by undergoing dramatic changes in shape that overcome inherent limitations. Future investigations into mechanisms that limit shape change, and regulate shape-dependent restriction points and the differentiation of new hair cells may contribute to the eventual development of treatments to promote cell replacement in the inner ear.

## Materials and Methods

### Ethics Statement

Experiments were performed according to protocols approved by the Animal Care and Use Committee of the University of Virginia (Protocol # 1835, NIH Animal Welfare Assurance Number A3245-01).

### Dissection of utricles

Swiss Webster mice were obtained from Charles River (Wilmington, MA) and sexually mature White Leghorn chickens from Slonaker Farm (Glengary, WV). Fertilized White Leghorn eggs (CBT Farms; Chestertown, MD) were incubated to hatching. Labyrinths were dissected from temporal bones in ice-cold DMEM/F-12 (Invitrogen, Carlsbad, CA), the utricles were isolated, and the roof and otoconia were mechanically removed under aseptic conditions.

### *In vitro* culture and wounding

Utricles were adhered to glass-bottom dishes (MatTek Corp, Ashland, MA) coated with CellTak (BD Biosciences, San Jose, CA). Wounding assays were conducted as previously described [14]. Briefly, 31 gauge stainless steel hypodermic needles (nominal internal diameter of 180 µM; Hamilton, Reno, NV) sharpened via electrolytic etching were pressed into utricles to make circular lesions in the center of the macula, and cells from within the lesion were excised with a tungsten needle without disturbing the underlying matrix (Fig. 4). Optimal culture conditions from our previous work and the literature were chosen for each of the species used in the study. Except where noted, utricles from mice were cultured in DMEM/F-12 containing 5% fetal bovine serum (FBS; Invitrogen), 3 µg/ml bromodeoxyuridine (BrdU; Sigma, St. Louis, MO), 0.25 µg/ml Fungizone (Invitrogen) and 10 µg/ml Ciprofloxacin (Bayer, Berlin, Germany). Utricles from chickens were cultured in M119 (with Earle's salts, 2,200 mg/l sodium bicarbonate, 0.69 mM l-glutamine, 25 mM HEPES; Invitrogen) supplemented with 10% FBS, 3 µg/ml BrdU, 0.25 µg/ml Fungizone and 10 µg/ml Ciprofloxacin.

To test for differences in culture conditions, chicken utricles were cultured in M119 supplemented with 0, 5 and 10% FBS and in DMEM/F12 (n = 4; Fig. S1; data not shown). Utricles from mice were also cultured in DMEM/F12 supplemented with 0, 5, or 10% FBS (n = 4; Fig. S1). Culture media and serum percentage had little effect on outcome, so data reported in the results is from experiments with optimal conditions. Utricles were fixed in 4% paraformaldehyde in PBS at the time points indicated in the results.

### *In vitro* spreading assays on flexible and rigid substrates

For experiments limiting macular spreading, utricles from E18.5–19.5 mice were incubated in thermolysin (0.5 mg/ml in DMEM/F-12; Sigma) at 37°C for 20 minutes, then transferred to DMEM/F12 containing 10% FBS, where the entire utricular epithelia (including the macula and non-sensory epithelium) were separated from the underlying matrix by microdissection. Delaminated epithelia were then plated on prepared coverglass-bottom culture dishes. Poly-lysine/fibronectin dishes were made by coating the coverglass with poly-lysine (5 µg/ml; Sigma) for 1 hour at 37°C, followed by fibronectin (100 µg/ml with 10% serum; Sigma) overnight at 37°C. Thin Matrigel coatings were made by spreading 2 µL of Matrigel (BD Biosciences) across the 10 mm diameter coverglass in the MatTek dish and allowing it to gel for 30 minutes at 37°C before hydration with DMEM/F-12. Thick Matrigel coatings were made by placing a 5 µL droplet of Matrigel in the center of the coverglass and allowing it to gel for 60 minutes prior to hydration with DMEM-F-12. To delineate the macula from the surrounding non-sensory epithelium, some cultures were incubated for 10 minutes in 15 µM FM 1–43 (Invitrogen), a styryl dye that selectively labels hair cells [55]. Images of the cultures were obtained after plating and at 1, 2 and 3 days with an Orca ER cooled CCD camera (Hamamatsu, Hamamatsu City, Japan) mounted on a Zeiss Axiovert 200 M (Carl Zeiss, Jena, Germany). After 3 days in culture, samples were fixed in Glyofixx (Thermo Scientific, Waltham, MA).

### Immunohistochemistry

Cultures were pre-incubated for 1 hour at room temperature (RT) in blocking solution: PBS containing 0.2% Triton X-100 (PBST) and 10% normal goat serum (NGS; Vector Laboratories, Burlingame, CA). Cultures to be labeled with anti-BrdU underwent a DNA denaturing treatment with DNAse I (0.5 kunitz/µL; Sigma) for 1 hour at 37°C or 1 N HCl for 20 minutes at RT before adding the blocking solution. Cultures were incubated in the appropriate primary antibodies in PBST with 2% NGS overnight, followed by 3 rinses in PBST and incubation with Alexa-conjugated secondary antibodies (1∶200, Invitrogen) and/or phalloidin (5 U/mL, Invitrogen) in PBST for 2 hours at RT. Utricles were rinsed in PBS 3 times and mounted in SlowFade (Invitrogen). Specimens were imaged with a Zeiss LSM 510 confocal microscope.

Rabbit anti-myosin VIIA (1∶200, Proteus Biosciences, Ramona, CA) or rabbit anti-calretinin (1∶1000; Chemicon, Temecula, CA) were used to label hair cells, and mouse anti-BrdU (1∶50; BD Biosciences) was used to label cells which had entered S-phase. To mark the apical borders of supporting cells, utricles were incubated with mouse anti-occludin (1∶200; Invitrogen), which labels tight junctions, or AlexaFluor-labeled phalloidin, which labels the filamentous actin in the circumferential belts at the adherens junctions.

**Measurement and analysis of the areas of apical cell outlines and BrdU incorporation** (See Methods S1)

## Supporting Information

Figure S1 **Extrinsic factors have little effect on wound closure and proliferation in chickens and mice.** (A) Graph on the left shows the time courses of wound closure for wounds made in P0 chicken utricles cultured with different amounts of serum added to the media. Wounds from utricles cultured with 5% and 10% serum were completely closed by 24 hours, whereas wounds from utricles cultured without serum were almost closed by 24 hours. Graph on right shows the number of BrdU+ nuclei at the lesion site 72 hours after wounding for the different concentrations of serum. There appear to be no significant differences in number of BrdU+ nuclei under the different culture conditions. (B) Graph on left shows the different time courses of wound closure for wounds made in P2 mouse utricles cultured with different amounts of serum added to the media. Wounds from utricles cultured with 5% and 10% serum were completely closed by 16 hours. In utricles cultured without serum, 82% of the wound was closed by 16 hours. Graph on right shows the number of BrdU+ nuclei at the lesion site 72 hours after wounding for the different concentration of serum. No differences in the number of BrdU+ nuclei were found under the different conditions.
(TIF)

Figure S2 **Supporting cells in utricles from P0 chickens enter S-phase and divide once within 48 hours after wound closure.** (A) Z-projected confocal image stack of the wound area in a P0 chicken utricle 48 hours after closure. Cell borders were labeled with phalloidin (green) and nuclei that entered S-phase were labeled with antibodies for BrdU (red). The wound was given 24 hours to close, then a blocker of microtubule polymerization, colchicine, was added to the media for an additional 48 hours to prevent cytokinesis. Colchicine treated cells were rounded with large BrdU+ nuclei, consistent with mitotic block. Scale bar, 100 µm. (B) Quantification of the number of BrdU+ nuclei within the 30,000 µm^2^ wound area in colchicine-treated or untreated control utricles. The number of BrdU+ nuclei in wounds of colchicine-treated utricles was roughly half that in control cultures, suggesting that supporting cells enter S-phase and divide once during the culture period.
(TIF)

Figure S3 **In wounded utricles from adult mice, many cells that undergo large shape changes are BrdU−.** Magnified views near the origin of the plots in Fig. 7 demonstrate that in P82 mice, there are many BrdU− data points (black circles) at the maximum y-values shown on the axis. In chickens and P2 mice, nearly all data points are BrdU+ (red circles) at similar values.
(TIF)

Figure S4 **Supporting cells from adult mice undergo the largest shape changes to close wounds.** A) Exponential curves fit to average apical cell areas as a function of their radial distance from the center of the wound. The averages were calculated by summing the apical areas of cells whose centroids fell within 1110 µm^2^ concentric annuli that were centered on the wound and dividing by the number of cells measured in each annulus (annuli became thinner with increased distance from the wound center in order to maintain the same area). The distance to the wound center for each average apical area was the outermost radius of its corresponding annulus. Near the center of the excision site, the average apical area of supporting cells in P82 mice was 1.8 times larger than in P2 mice and 2.4–4 times larger than in chickens. Moving away from the wound center, supporting cell apical areas decrease and become similar to their *in vivo* values (blue lines).
(TIF)

Table S1 **Mean open areas of wounds made in utricles from mice and chickens of different ages.**
(DOCX)

Table S2 **Average BrdU+ nuclei in wounds in utricles from mice and chickens of different ages.**
(DOCX)

Methods S1 **Measurement and analysis of the areas of apical cell outlines and BrdU incorporation.**
(DOCX)
