# Supplementary figures and images for: Variations in Shape-Sensitive Restriction Points Mirror Differences in the Regeneration Capacities of Avian and Mammalian Ears
Source: PLoS One. 2011 Aug 31;6(8):e23861. doi: 10.1371/journal.pone.0023861 (PMC3166124; doi:10.1371/journal.pone.0023861)

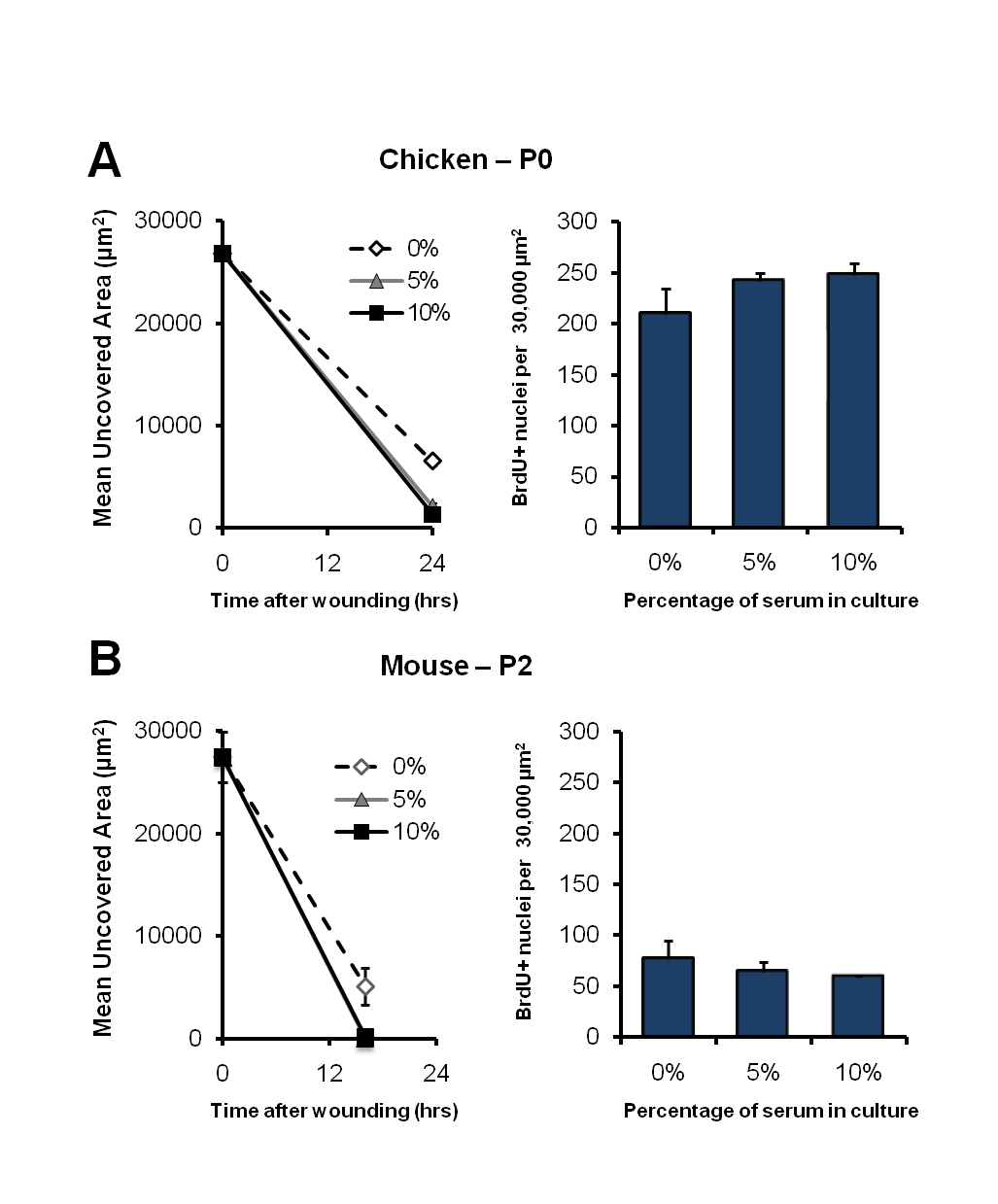

Supplement: Figure S1 — Extrinsic factors have little effect on wound closure and proliferation in chickens and mice. (A) Graph on the left shows the time courses of wound closure for wounds made in P0 chicken utricles cultured with different amounts of serum added to the media. Wounds from utricles cultured with 5% and 10% serum were completely closed by 24 hours, whereas wounds from utricles cultured without serum were almost closed by 24 hours. Graph on right shows the number of BrdU+ nuclei at the lesion site 72 hours after wounding for the different concentrations of serum. There appear to be no significant differences in number of BrdU+ nuclei under the different culture conditions. (B) Graph on left shows the different time courses of wound closure for wounds made in P2 mouse utricles cultured with different amounts of serum added to the media. Wounds from utricles cultured with 5% and 10% serum were completely closed by 16 hours. In utricles cultured without serum, 82% of the wound was closed by 16 hours. Graph on right shows the number of BrdU+ nuclei at the lesion site 72 hours after wounding for the different concentration of serum. No differences in the number of BrdU+ nuclei were found under the different conditions. (TIF) [file pone.0023861.s001.tif]

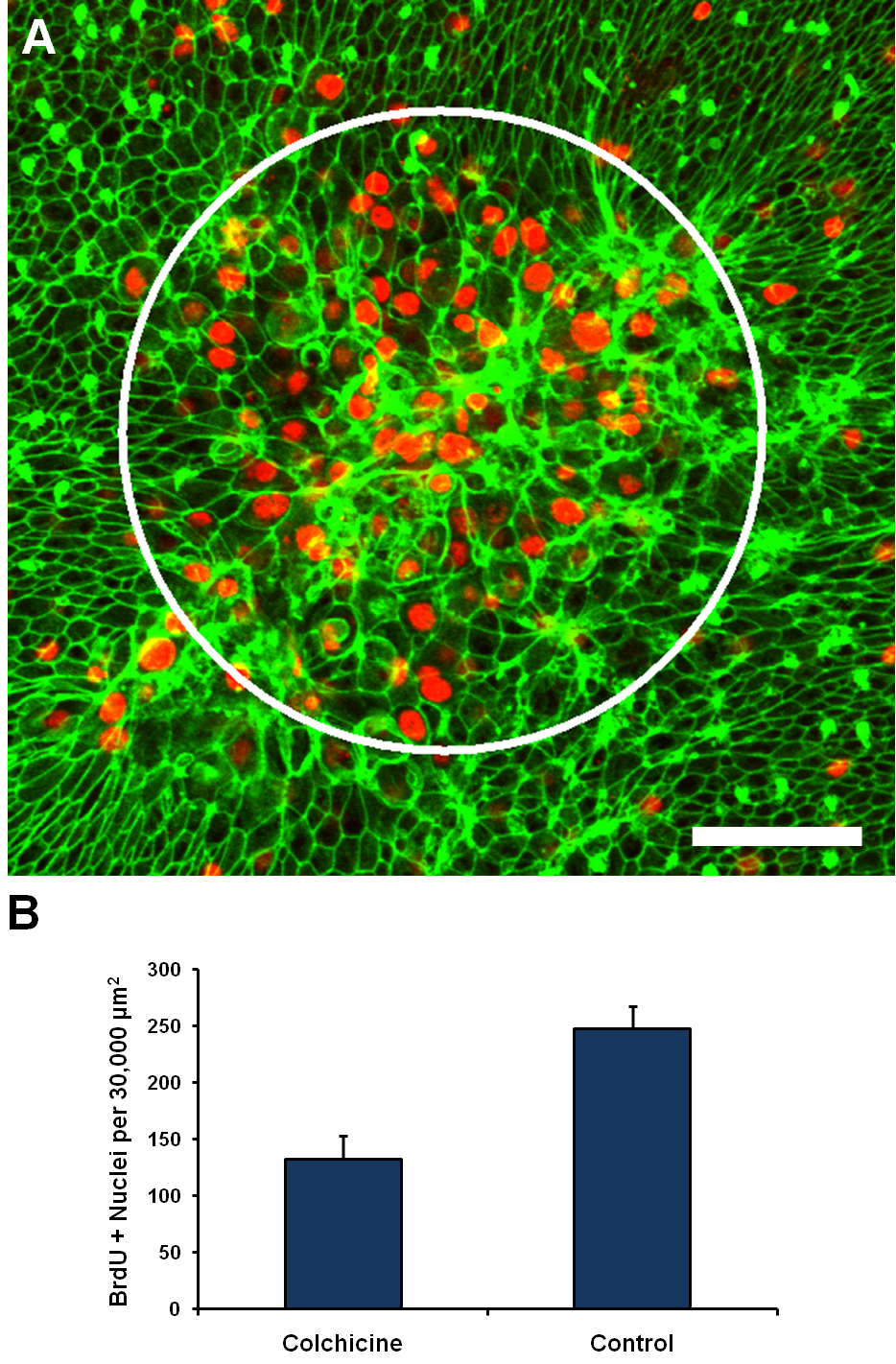

Supplement: Figure S2 — Supporting cells in utricles from P0 chickens enter S-phase and divide once within 48 hours after wound closure. (A) Z-projected confocal image stack of the wound area in a P0 chicken utricle 48 hours after closure. Cell borders were labeled with phalloidin (green) and nuclei that entered S-phase were labeled with antibodies for BrdU (red). The wound was given 24 hours to close, then a blocker of microtubule polymerization, colchicine, was added to the media for an additional 48 hours to prevent cytokinesis. Colchicine treated cells were rounded with large BrdU+ nuclei, consistent with mitotic block. Scale bar, 100 µm. (B) Quantification of the number of BrdU+ nuclei within the 30,000 µm2 wound area in colchicine-treated or untreated control utricles. The number of BrdU+ nuclei in wounds of colchicine-treated utricles was roughly half that in control cultures, suggesting that supporting cells enter S-phase and divide once during the culture period. (TIF) [file pone.0023861.s002.tif]

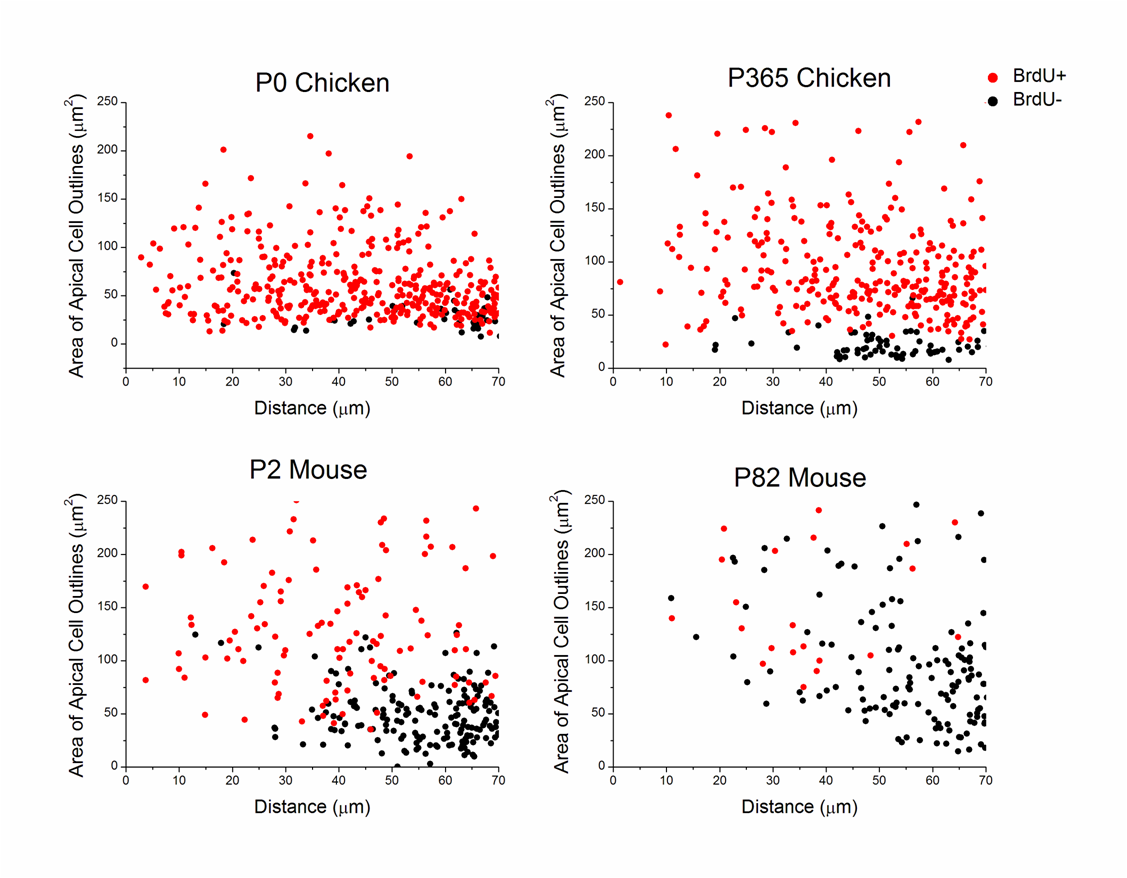

Supplement: Figure S3 — In wounded utricles from adult mice, many cells that undergo large shape changes are BrdU−. Magnified views near the origin of the plots in Fig. 7 demonstrate that in P82 mice, there are many BrdU− data points (black circles) at the maximum y-values shown on the axis. In chickens and P2 mice, nearly all data points are BrdU+ (red circles) at similar values. (TIF) [file pone.0023861.s003.tif]

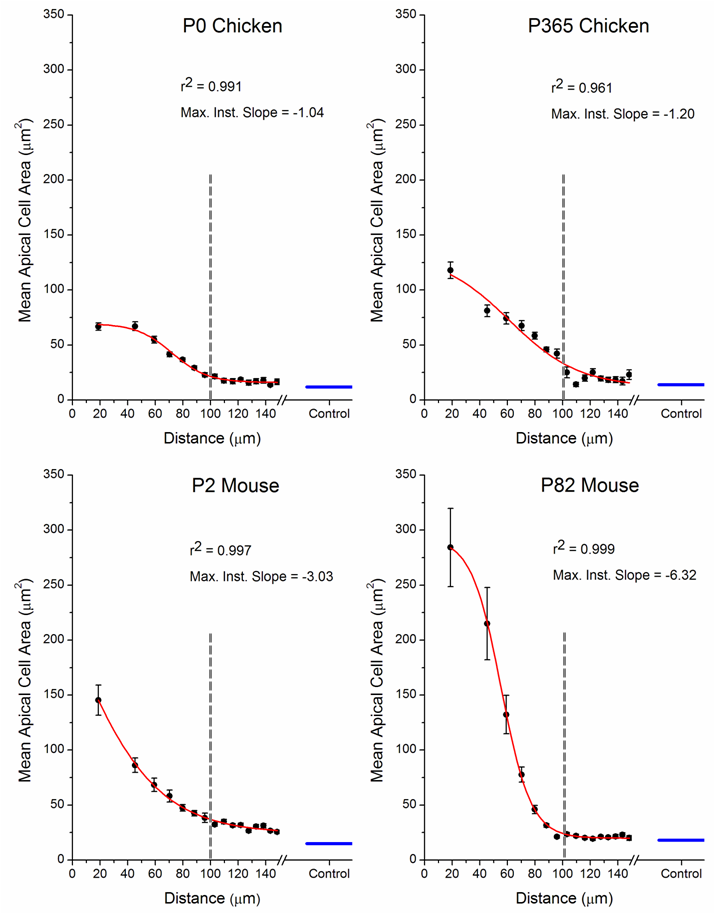

Supplement: Figure S4 — Supporting cells from adult mice undergo the largest shape changes to close wounds. A) Exponential curves fit to average apical cell areas as a function of their radial distance from the center of the wound. The averages were calculated by summing the apical areas of cells whose centroids fell within 1110 µm2 concentric annuli that were centered on the wound and dividing by the number of cells measured in each annulus (annuli became thinner with increased distance from the wound center in order to maintain the same area). The distance to the wound center for each average apical area was the outermost radius of its corresponding annulus. Near the center of the excision site, the average apical area of supporting cells in P82 mice was 1.8 times larger than in P2 mice and 2.4–4 times larger than in chickens. Moving away from the wound center, supporting cell apical areas decrease and become similar to their in vivo values (blue lines). (TIF) [file pone.0023861.s004.tif]
