# Supplementary material for: Variations in Shape-Sensitive Restriction Points Mirror Differences in the Regeneration Capacities of Avian and Mammalian Ears
Source: PLoS One. 2011 Aug 31;6(8):e23861. doi: 10.1371/journal.pone.0023861 (PMC3166124; doi:10.1371/journal.pone.0023861)
Supplement: Table S1 — Mean open areas of wounds made in utricles from mice and chickens of different ages. (DOCX) [file pone.0023861.s005.docx]

**TABLE S1: Mean open areas of wounds made in utricles from mice and chickens of different ages**

|  | **Open wound area (µm^2^)** | | | |
| --- | --- | --- | --- | --- |
| **Time after wounding** | **0 hrs** | **24 hrs** | **48 hrs** | **72 hrs** |
| P0 chickens | 2.7 x 10^4^ ± 0.1 x 10^4^ μm^2^  (n = 6) | 1.3 x 10^3^ ± 0.7 x 10^3^ μm^2^ (n = 6) | Closed (0 μm^2^)  (n=4) |  |
| P365 chickens | 2.7 x 10^4^ ± 0.1 x 10^4^ μm^2^  (n = 6) | 615 ± 310 μm^2^  (n = 6) | Closed (0 μm^2^)  (n=4) |  |
| P2 mice | 2.7 x 10^4^ ± 0.2 x 10^4^ μm^2^  (n = 7) | Closed (0 μm^2^)  (n=4) |  |  |
| P16 mice | 2.7 x 10^4^ ± 0.2 x 10^4^ μm^2^  (n = 7) | 1.6 x 10^4^ ± 0.2 x 10^4^ μm^2^ (n = 9) | 3.7 x 10^3^ ± 1.7 x 10^3^ μm^2^  (n = 6) | Closed (0 μm^2^)  (n=7) |
| P82 mice | 2.7 x 10^4^ ± 0.2 x 10^4^ μm^2^  (n = 7) | 1.8 x 10^4^ ± 0.2 x 10^4^ μm^2^ (n = 5) | 1.2 x 10^4^ ± 0.5 x 10^4^ μm^2^ (n = 5) | 575 ± 575 μm^2^  ( n = 6) |
