# Supplementary material for: Variations in Shape-Sensitive Restriction Points Mirror Differences in the Regeneration Capacities of Avian and Mammalian Ears
Source: PLoS One. 2011 Aug 31;6(8):e23861. doi: 10.1371/journal.pone.0023861 (PMC3166124; doi:10.1371/journal.pone.0023861)
Supplement: Table S2 — Average BrdU+ nuclei in wounds in utricles from mice and chickens of different ages. (DOCX) [file pone.0023861.s006.docx]

**TABLE S2: Average BrdU+ nuclei in wounds in utricles from mice and chickens of different ages**

|  | **Average number of BrdU+ nuclei in 30,000 µm^2^ wound area** | | | | | |
| --- | --- | --- | --- | --- | --- | --- |
| **Time after wounding** | | **24 hrs** | **48 hrs** | **72 hrs** | **96 hrs** | **120 hrs** |
| P0 chickens | | 6 ± 1 (n = 7) | 199 ± 23 (n = 4) | 247 ± 13 (n = 6) |  |  |
| P365 chickens | | 2 ± 1 (n = 7) | 235 ± 16 (n = 4) | 257 ± 23 (n = 6) |  |  |
| P2 mice | | 13 ± 4 (n = 4) | 34 ± 3 (n = 6) | 64 ± 10 (n = 9) | 79 ± 18 (n = 7) | 65 ± 16 (n = 5) |
| P16 mice | | 1 ± 1 (n = 9) | 10 ± 3 (n = 6) | 11 ± 3 (n = 7) | 42 ± 8 (n = 4) | 44 ± 6 (n = 4) |
| P82 mice | | 0 (n = 5) | 2 ± 1 (n = 5) | 16 ± 6 (n = 3) | 12 ± 2 (n = 6) | 29 ± 3 (n = 5) |
