# Supplementary material for: Variations in Shape-Sensitive Restriction Points Mirror Differences in the Regeneration Capacities of Avian and Mammalian Ears
Source: PLoS One. 2011 Aug 31;6(8):e23861. doi: 10.1371/journal.pone.0023861 (PMC3166124; doi:10.1371/journal.pone.0023861)
Supplement: Methods S1 — Measurement and analysis of the areas of apical cell outlines and BrdU incorporation. (DOCX) [file pone.0023861.s007.docx]

**METHODS S1**

**Measurement and analysis of the areas of apical cell outlines and BrdU incorporation**

Measurements of the area of apical cell outlines and counts of BrdU+ nuclei were performed manually using Metamorph or Image J and tabulated in OriginPro. Outlines were traced around the apical borders of cells in images of samples labeled with anti-occludin or phalloidin, and the areas of the apical cell outlines were recorded. If any portion of a BrdU+ nucleus fell within the outline of an individual cell, that cell was also recorded as BrdU+, so that area and BrdU information for the same cell could be generated.
